# Barriers to raising taxes on tobacco products in Uganda: a political economy analysis

**DOI:** 10.1093/heapol/czaf098

**Published:** 2025-11-28

**Authors:** Henry Zakumumpa, Ligia Paina, Eric Ssegujja, Richard Ssempala, Freddie Ssengooba

**Affiliations:** School of Public Health, Makerere University, New Mulago Hill Road, PO Box 7062, Mulago, Kampala, Uganda; Bloomberg School of Public Health, Johns Hopkins University, 615 N. Wolfe Street, Room E8543, Baltimore, MD 21205, United States; School of Public Health, Makerere University, New Mulago Hill Road, PO Box 7062, Mulago, Kampala, Uganda; School of Economics, Makerere University, Plot 51 Pool Road, Makerere Main campus, Kampala, Uganda; School of Public Health, Makerere University, New Mulago Hill Road, PO Box 7062, Mulago, Kampala, Uganda

**Keywords:** tobacco control, health policy, health taxes, political economy, non-communicable diseases

## Abstract

Raising taxes on tobacco is considered the most effective measure for reducing tobacco consumption. Although Uganda ratified WHO’s Framework Convention on Tobacco control which recommends levying taxes on tobacco products by up to 75% of their retail price, in Uganda taxes on tobacco stagnated at 35% between 2017 and 2024. There is little in-depth research interrogating the political economy underpinning tobacco tax policy in Uganda. The aim of this study is to apply political economy analysis in exploring barriers to implementing WHO’s recommended tobacco tax rates in Uganda. Our qualitative study entailed key informant and in-depth interviews with 34 purposively selected participants. Data were analysed by thematic approach. Tobacco industry narratives are dominant among policy elite with a strongly entrenched notion that raising taxes will bring economic harm such as ‘killing off’ the tobacco industry and by implication diminish government tax revenue. Participants identified tobacco industry interference in tobacco tax policy in Uganda through both ‘soft’ tactics such as sustained lobbying of policy elite in the executive and legislative arms of government and ‘hard’ tactics through litigation. Contrary to recommendations of having a ‘single spine’ or uniform tax on tobacco, Uganda continues to implement a differential tax structure for tobacco products. The paucity of non-industry-funded—research on effects of raising tobacco taxes was observed while the attrition of civil society champions in advocacy campaigns for raising taxes ensured that there was no sustained counterbalance to the tobacco industry in Uganda which the later exploited to promote the narrative that taxes needed to be maintained at a low level where they would not cause ‘economic harm’. Our findings highlight the need for strengthening civil society advocacy in order to sustain the momentum on raising tobacco taxes in Uganda.

Key messages

- Despite Uganda’s commitment in the Framework Convention on Tobacco Control of progressively raising taxes on tobacco to align with inflation and other changing macro-economic variables, there was no increase in tobacco taxes between 2017 and 2024.
- Over the past two decades, there has been an attrition of champions in the civil society organizations coalition for raising tobacco taxes. This has left a vacuum exploited by the tobacco industry lobby in its quest to maintain the status quo in tobacco tax rates.
- Tobacco industry misinformation dominates among tax authorities with a strongly entrenched notion that raising taxes will bring economic harm such as ‘killing off’ the tobacco industry and by implication government tax revenue.
- We identified industry interference in tobacco tax policy in Uganda through both ‘soft’ tactics such as sustained lobbying of policy elite in the executive and legislative arms of government and ‘hard’ tactics through threats of litigation.

## Introduction

Globally, tobacco is the leading cause of preventable death (Tauras 2024). Tobacco use is the only risk factor common to all the major non-communicable diseases (NCDs) that include cancers, cardiovascular diseases and respiratory illnesses (Kabwama et al. 2016). Low and middle-income countries (LMICs) are confronting an escalating epidemic of NCDs (Katzmarzyk et al. 2022) with tobacco use recognized as a significant contributor to this rising epidemic. The role of transnational companies in promoting tobacco and undermining regulatory schemes in LMICs has become a major problem for mitigating NCDs (Egbe et al. 2022).

To address the epidemic of tobacco-associated illnesses across the globe such as lung cancer, the World Health Organization (WHO) adopted the Framework Convention on Tobacco Control (FCTC) in 2003 (Egbe et al. 2022). The FCTC contains a range of evidence-based tobacco control strategies key among which are fiscal and price measures such as tax on tobacco which were passed in 2014 (Spencer et al. 2024). Among the key policy recommendations of the FCTC, is that taxes on tobacco constitute 75% of the retail price of tobacco products (WHO 2015). Some high-income countries such as Australia have raised excise duty on cigarettes to meet WHO’s recommended minimum threshold (Wilkinson et al. 2019). However, many LMICs continue to fall short of the minimum recommended tax as a proportion of the retail price of tobacco products despite having ratified the FCTC.

Raising taxes—and reducing affordability of tobacco is an evidence-based measure in reducing consumption particularly among young people who are more price sensitive (Sheikh et al. 2025).

The WHO recommends a 75% tax on the retail price of tobacco which is underpinned by evidence that proves that at that rate affordability reduces among key sub-groups (WHO 2015). Raising taxes on tobacco would have particularly remarkable effects on consumption in LMICs particularly with respect to low-income smokers who are more price sensitive (Nargis et al. 2021) and more so in Uganda which has a significant part of its population living below the poverty line (Datzberger 2018). In addition, in LMICs, younger people use tobacco products more than older individuals and hence fiscal and price measures have immense promise as a tobacco control measure (Londani and Oladimeji 2024). In Uganda, 13–15 years olds have the highest rates of tobacco use at ∼19% prevalence compared with 12% among adult men (Daama et al. 2023). In Uganda, males use tobacco more than females. The Global Youth Tobacco Survey of 2018 revealed that in Uganda, more boys (11.7%) compared with girls (9.2%) used tobacco while more men (11.3%) than women (2.6%) used tobacco according to the 2023 Global Adult Tobacco Survey with respect to Uganda (GATS 2023).

Uganda signed the FCTC in 2007 which was followed with the enactment of a comprehensive tobacco control law in 2015 (Lukwata et al. 2023). Although the tobacco control law in Uganda provides for price and tax measures, it stops short of setting a lower limit on tax on tobacco as recommended in the FCTC (Basaza et al. 2017).

In Uganda, tax rates on cigarettes currently stand at 35% which is less than half of the recommended minimum tax by the WHO. Perhaps unsurprisingly, taxes on tobacco in Uganda are yet to significantly dent consumption (Ntale and Kasirye 2018). Indeed studies show that despite taxation on tobacco in Uganda consumption has continued unabated since 2015 when the tobacco control law was passed (Filby et al. 2024). Matters are not helped by Uganda implementing different tax rates for different tobacco products contrary to the WHO recommendation of having a ‘single spine’ tax structure or uniform tax on all tobacco products. For instance, imported tobacco products attract significantly higher taxes compared with ‘locally manufactured’ ones (Filby et al. 2024). A recent civil society report from the Uganda Tobacco-Taxation Coalition (UTTC) revealed that Uganda had not raised taxes on tobacco between 2017 and 2024 despite a 29% increase in inflation over the same period as well as other changes in macro-economic indicators (New Vision 2025). The implication is that once taxes on tobacco are not raised progressively in line with inflation, products such as cigarettes become more affordable (Filby et al. 2024). In contrast, Rwanda, a neighbouring country with a similar low-income classification as Uganda, recently adopted higher taxes on tobacco signalling that political will may be influential (The Inspirer 2025).

The tobacco industry in Uganda has pushed back against raising taxes citing ‘negative’ impacts on the tobacco value chain. It is estimated that over 75 000 small-holder farmers depend on tobacco leaf farming as a livelihood in the West Nile sub-region of Northern Uganda and Kanungu district in Western Uganda (Tobacco Atlas 2025). British American Tobacco Uganda (BATU) alone reportedly contracts over 18 000 small-holder tobacco farmers. Tobacco leaf growing in Uganda is labour intensive and uses largely traditional farming methods. In 2022, it was reported that Uganda produced 33 000 tons of tobacco leaf principally from the districts of Arua and Kanungu in Northern and Western Uganda, respectively (Tobacco Atlas 2025). The principal workforce cadres in tobacco leaf production in Uganda include leaf classifiers, supervisors with manual tasks entailing grading, blending, and preparing tobacco leaves. In 2013, BATU stopped cigarette production in Uganda and moved this to neighbouring Kenya (Tobacco Tactics 2025).

Most studies on tobacco taxation in Uganda have been economic studies assessing the impact of taxation on tobacco consumption in Uganda (Obwona et al. 2005, Ntale and Kasirye 2018, Chelwa and van Walbeek 2019).

There is little in-depth research exploring the political economy underpinning tobacco tax policy in Uganda and other low-income countries (Bump and Reich 2013, Elliott et al. 2020, Hoe et al. 2021).

The aim of this study is to apply political economy analysis (PEA) in exploring barriers to implementing WHO’s recommended tobacco tax rates in Uganda.

## Materials and methods

As a part of a WHO/Alliance for Health Policy and Systems research grant aimed at development of a series of analytical country case studies to better understand the political economy of health taxes in LMICs (WHO 2021), we implemented a qualitative exploratory research design to understand barriers to raising taxes on tobacco from the perspective of relevant local stakeholders in Uganda.

### Political economy analysis lenses

Our study utilized PEA lenses (Gill and Benatar 2020) in exploring barriers to raising taxes on tobacco in Uganda. Within the suite of PEA approaches, we broadly drew upon the political settlements analysis (PSA) framework which we adapted from governance literature and applied to interrogating the political economy of health taxes in Uganda. PSA posits that ‘policy choices’ are shaped by contests among contending elite groups over ‘material interests’ and that these contests among ‘elite’ groups are often settled through complex policy compromises (Khan 2018). PSA maintains that public policy outcomes are shaped by fierce contests among competing interests of dominant actors in a polity and that ‘bargaining outcomes’ are often reached when their rival interests are accommodated in a mutually agreed ‘tacit settlement’ reached to avoid conflict (Khan 2018). Proponents of PSA argue that formal state institutions with decision-making power such as Ministries of Finance which make economic policy are influenced by powerful interests in reaching governance decisions. Kelsall (2018) have described PSA thus ‘a political settlement is a tacit agreement among powerful groups about the rules of the political and economic game, that keeps the peace by providing opportunities for those groups to secure a distribution of benefits (such as resources, rights, and status) that they find acceptable’. PSA has a strong emphasis on the analysis of organizations and institutions that mediate conflicting interests among contending ‘elite groups’ and the distribution of power in formal institutions. This includes a comparative analysis of the balance of power in terms of ‘organizational capability’ of the competing ‘elite’ groups in shaping public policy.

We utilized the PSA framework in four ways. We applied the framework in understanding the underlying domestic context in terms of the actors, interests and institutions that shape tobacco tax policy in Uganda. In Table 2 we show how the PSA framework helped us explore the political economy of tobacco tax policy in Uganda. We utilized the framework to identify study participants from the PSA dimension of ‘contending’ interest groups such as the Uganda tobacco industry lobby on one hand and the UTTC on the other hand as well as mediating formal institutions with decision-making power such as the Uganda Ministry of Finance which makes tobacco tax policy and the Uganda Revenue Authority (URA) which determines tobacco tax rates. Secondly, the PSA framework informed the development of our interview guides (such as in probing the interests of varied actors and their tactics). The PSA guided our qualitative data analysis and informed the development of our initial coding scheme. Fourthly, in the discussion section we draw upon the PSA framework in our overall synthesis and interpretation of the five themes presented in the Results section.

### Data collection

#### In-depth interviews

In line with the PSA framework, we identified influential actors in tobacco tax policy making in Uganda (such as high-level tax officials), institutions (such as the Ministry of Finance) through the process of literature search which we describe below. Hence, we purposively sampled 36 diverse local stakeholders knowledgeable about tobacco tax policy and the underpinning political economy context. We included individuals from a local civil society coalition known as the UTTC with institutional memory of past campaigns for raising taxes on tobacco in Uganda and who could share insights and experiences around multi-sectoral partnerships on the subject over the past two decades in Uganda (New Vision 2025). We also interviewed technocrats from the Ministry of Health, the Ministry of Finance and officials from URA who could share retrospective perspectives on trends in taxes on tobacco in Uganda since 2015, as well as officials from the WHO Uganda country office, local public health academics, and specialist researchers knowledgeable on the economics of tobacco control at the Uganda-based Centre for Tobacco Control in Africa. Table 1 shows the organizational affiliation of study respondents.

**Table 1.** Respondent summary

Category of participants	Number of respondents
Ministry of Health Officials	03
Ministry of Finance and Uganda Revenue Authority officials	06
World Health Organization—Uganda officials	02
Public health researchers	04
Ugandan civil society actors	16
Continental Tobacco control bodies	02
Total	34

On average, the in-depth interviews (IDIs) lasted between 40 and 60 min. The face-to-face interviews were conducted by three of the authors (H.Z., E.S., and R.S.) between December 2023 and February 2024.

#### Desk reviews

We supplemented our predominant primary data collection component with desk reviews of the literature on tobacco industry tactics in Uganda as it relates to tobacco tax policy. Broadly, in our desk reviews we followed recommended procedures for literature review (McKee and Britton 1997). We conducted an environmental scan of relevant published and grey literature on the dominant tobacco industry players in Uganda, principally British American Tobacco. Our search terms included ‘tobacco’ OR ‘cigarette’ AND ‘tax*’ AND Uganda. We searched web of science, Google scholar and Scopus. Our search was from February 2015, the year when Uganda passed a tobacco control law to April 2025. The inclusion criteria was such that we included the published and grey literature that reported empirical research on the subject of the Uganda tobacco industry and tobacco tax policy making processes in Uganda. We excluded opinion pieces, commentaries and editorials. Secondly, we searched the websites of research bodies on tobacco industry tactics (e.g. www.tobaccotactics.org and https://ash.org/). Our literature search aimed at identifying strategies by the tobacco industry in Uganda aimed at influencing tobacco tax policy (e.g. through lobbying or corporate social responsibility schemes).

### Data analysis

Data were analysed in two parts; (i) thematic content analysis of the documents we identified from the literature search described earlier, (ii) thematic analysis of the coded data from our key informant and IDIs.

### Desk reviews

We conducted a thematic content analysis of the documents identified through the desk review described earlier. Our thematic content analysis aimed at synthesizing the identified strategies by the tobacco industry in Uganda in its quest for a favourable tobacco tax regime. We generated a master data extraction file indicating the name of the document, the author, the year of publication with respect to the tobacco industry ‘soft’ tactics (e.g. lobbying tobacco tax policy makers) or ‘hard tactics’ (such as use of litigation) as initial codes to facilitate thematic analysis. The second step entailed extracting large text verbatim excerpts from the documents and categorizing them under either of the two categories using interpretive analysis which was guided by the PSA framework (e.g. contending ‘elite’ groups, interests, formal state institutions and other key elements shown in Table 2). After the initial categorization, the material was re-read and large text verbatim excerpts were subsequently condensed to find common patterns and themes from across the identified documents.

**Table 2.** Political settlements mapping of tobacco tax policy in Uganda

Key elements	Identifying actors	Summary description
Contending ‘elite’ groups	Tobacco industry Uganda Tobacco-Taxation Coalition	The two principal contending groups with respect to raising tobacco taxes entail the tobacco industry in Uganda whereby British American Tobacco Uganda is the dominant player. Civil society organizations in Uganda advocating for raising taxes on tobacco coalesced under a coalition—Uganda Tobacco-Taxation Coalition.
Interests	Tobacco industry	Low taxes that ensure products are affordable thereby enhancing profitability.
	Government of Uganda	Protecting tax income accruing from the tobacco industry and its secondary benefits to the Uganda economy.Protecting domestic industries from foreign competition.
	Uganda Tobacco-Taxation Coalition	Raising tobacco taxes to reduce affordability for younger and low-income smokers.
Identification of state institutions	National parliament Ministry of Finance Uganda Revenue Authority Courts of Judicature of Uganda	There are a set of institutions in Uganda with decision-making power on tobacco tax policy in all three arms of government of the legislature, executive, and judiciary.
Distribution of organizational power	Legislative Tobacco tax planning Tobacco tax administration Judicial	The National Parliament approves the annual national budget including annual tax proposals of Government of Uganda. Ministry of Finance is charged with tax policy making including for tobacco products. Uganda Revenue Authority assesses tobacco tax rates and overall tax administration in this regard. The Courts of Judicature in Uganda adjudicate tax disputes involving petitions by private companies against Government of Uganda.
‘Mass mobilization capability’	Tobacco industry	The tobacco industry demonstrates formidable mobilization capability in varied ways including the use of ‘front groups’ such as tobacco farmers and retail traders, ability to institute legal suites, leveraging networks with Ministry of Trade officials in their ‘low tax’ agenda.
	Uganda Tobacco-Taxation Coalition	Attrition of champions weakened sustained mobilization capability.

### Qualitative interviews

We adopted the qualitative data analysis procedures recommended by Huberman and Miles (1994). More specifically, we used a ‘framework analysis’ approach (Gale et al. 2013). Although our qualitative data analysis was iterative, we followed four major steps. An initial coding scheme was developed as broadly guided by the key elements of the PSA framework (e.g. contending ‘elite groups’, interests, formal institutions, organizational capability). As a first step, three investigators (H.Z., E.S., and R.S.) read through the interview transcripts separately to identify common themes emerging from the interview responses with respect to exploring barriers to implementing WHO’s recommended tobacco tax rates in Uganda. In the second stage, the investigators compared the themes emerging from the interviews against those in the PSA framework-informed initial coding scheme. In the third stage, the initial codes were then methodically merged under the five emergent themes of the study (i) dominant industry narratives of the ‘economic harm’ of raising tobacco taxes, (ii) attrition of local champions from the tobacco-taxation advocacy coalition, (iii) interference in tobacco tax policy by vested interests). Hence, we used a hybrid approach of inductive and deductive coding and theme development (Fereday and Muir-Cochrane 2006). Four investigators (H.Z., E.S., R.S., and F.S.) sought consensus in the interpretation and the assignment of codes and themes with respect to barriers to implementation of WHO’s recommended tobacco tax rates in Uganda. Interviewee data were triangulated with other data sources such as the desk reviews (e.g. published reports of Uganda tobacco industry tactics). The findings emerging from desk reviews were categorized them under the five themes presented in the Results section.

The fourth and final step was that of ‘overall synthesis and interpretation’ which involved all five co-authors (H.Z., E.S., R.S., L.P., and F.S.).

## Results

The results are presented according to five themes relating to (i) dominant industry narratives of the ‘economic harm’ of raising tobacco taxes, (ii) attrition of local champions from the tobacco-taxation advocacy coalition, (iii) interference in tobacco tax policy by vested interests, (iv) paucity of localized evidence on the effects of higher taxes on tobacco, (v) differential tax structure for tobacco products.

In line with the PSA, we identified various actors such as ‘policy elite’ who broadly refer to government officials with decision-making power such as high-level technocrats at the Uganda Ministry of Finance who plan tax policy, tax officials from the URA who are charged with tax administration as well as members of parliament in the national assembly who approve annual government tax proposals. The tobacco industry in Uganda is wholly owned by private firms with no government shareholding in any of the entities. The leading tobacco industry player in Uganda is BATU a transnational entity with a 51.7% market share (Tobacco Tactics 2025). BATU began operations in Uganda in the 1920s during British colonial rule (Tobacco Tactics 2025). However, there are other notable locally based industry players such as Leaf Tobacco and Commodities and Continental Tobacco Uganda who collectively push for low taxes on tobacco (Ntale and Kasirye 2018). On the other hand, ‘contending’ groups advocating for higher taxes on tobacco in Uganda include civil society coalitions such as the UTTC and public health researchers (University of Bath 2025). Table 2 shows our PSA mapping of tobacco tax policy making in Uganda from the lens of five key elements from the PSA framework (e.g. contending elite groups, interests, institutions).

### Dominant industry narratives of ‘economic harm’ of raising tobacco taxes

It emerged that there was a deeply entrenched notion among policy elite in Uganda that raising taxes would cause economic harm such as ‘killing off’ the tobacco industry and by implication, government tax revenue. Our IDIs revealed a widely held perception particularly among key actors at the Ministry of Finance and local tax revenue authorities that taxes on tobacco in Uganda could not be raised much higher than the prevailing rate of 35% due to fears that it would hurt government tax revenue by depressing demand for tobacco products. Local tax officials pointed to BATU’s withdrawal from manufacturing cigarettes in Uganda in favour of Kenya which the latter framed as arising from an ‘unfavourable’ tax regime.

As you may recall BAT used to have a huge cigarette manufacturing factory here in Uganda which they shifted to Kenya due to high taxation on locally manufactured cigarettes. Did you notice that? As a country we lose out. We have to appreciate that we operate in a competitive region and BAT has options in the region. They can decide to move to Kenya and manufacture from there and still move their products here in Uganda. So, when you think about such scenarios, then you don't want to disadvantage Uganda as an economy. I agree that public health is very important but also we need our economy to be vibrant. We need to create jobs here. (Tax official_03)

We observed that Ugandan policy elite and tax authorities frequently parrot industry ‘talking points’ borne out of years of misinformation by the industry that taxes could not be raised beyond an imaginary threshold or else the tobacco industry would fail and government would lose tax income. Although we did not find evidence directly linking the tobacco industry in influencing Ugandan policy elite we discerned this indirectly through interviews. For instance, it emerged that the tobacco industry in Uganda engages in protracted and sustained lobbying of policy elites which was revealed by their regurgitation of industry ‘talking points’ around the need for a ‘safe’ tax threshold that would not ‘kill off’ the industry. In addition, policy elite acknowledged regular face-to-face meetings with representatives from the industry such as during annual national budget stakeholder meetings. The narrative promoted by the industry around the notion of ‘economic harm’ associated with adopting a higher tax regime on tobacco products was evident in our interviews. Officials from the Ministry of Finance and Uganda tax authorities alluded to the need for striking a balance during tax policy making to ensure that the industry is protected from ‘failing’ and by extension that government tax revenues are secured.

We are perpetually grappling with striking a balance between revenue generation and public health objectives. We are always thinking of local industries. When you are charging tax rates, you need to be conversant with the possible consequences. You don't want to kill the economy. You don't want to kill the manufacturers. They complain each time you keep increasing tax rates. So we are torn between revenue generation and health. The tax rates should not be in such a way that the industry is totally wiped out because the industry has broader benefits to the economy. I appreciate the reasoning from a health perspective but have you thought about the Ugandans jobs we would lose, the tobacco farmers? As a country we need to strike a balance. (KI_Ministry of Finance Official_02)

Uganda has a low tax-to-gross domestic product ratio of 13% that is low even by standards of low-income countries in Africa (Mawejje and Munyambonera 2016). As such, Uganda is dependent on a dozen institutional ‘large tax payers’ mostly multi-national companies (Ntale and Kasirye 2018). BATU was regarded as one of the ‘large tax payers’ even when the Ugandan economy has expanded and diversified considerably over the past two three decades (Ntale and Kasirye 2018).

In addition, there were deeply entrenched notions among policy elite of the importance of the tobacco industry to the local Ugandan economy in terms of providing jobs to retail businesses along the tobacco value chain. There was frequent mention of the livelihoods of local tobacco leaf farmers particularly in the West Nile Region of Northern Uganda and Bunyoro sub-region in Western Uganda (Nakamatte et al. 2015).

The tobacco industry in Uganda posits that significant tax increases could culminate in increased cross-border smuggling of un-taxed tobacco products. Kenya which borders Uganda to the east is a leading import market of cigarettes consumed in Uganda.

We have porous borders. Cigarettes are often smuggled from neighboring countries into Uganda. It is very dangerous but very lucrative to hide cigarette boxes in the boot of an old Toyota and cross the border from Kenya into Uganda. It brings windfall profits. The profits are so high that they can actually continue attempting as much as possible to smuggle the cigarettes. So we need to be mindful about what will happen if we actually increase the tax rates. The level at which we increase taxes we must study it and know the effects such that we do not push people into illicit trade. (Ministry of Finance official, 02)

Desk reviews revealed that BATU uses legal tactics and litigation as push back against tax hikes on tobacco products (Jackson et al. 2021). BATU has repeatedly sued the URA over tax disputes, with several cases recorded at the Tax Appeals Tribunal of Uganda and the East African Court of Justice (EACJ 2017, TAT 2022). The astute selection of chairs of the governing board of BATU involved carefully selected former cabinet ministers in the Uganda government or influential former heads of government parastatals with vast high-level connections to Uganda’s political elite. A published investigative report has alleged the use of bribes to local policy elite as part of pushback against Uganda’s adoption of higher taxes on tobacco or ‘unfriendly’ laws (Jackson et al. 2021).

### Attrition of local champions from the tobacco-taxation advocacy coalition

Our interviews revealed that local tobacco control advocates have engaged in a decades-old tobacco-taxation advocacy campaign which has suffered attrition over time of key champions. The loss of these champions was said to have resulted in loss of personal ties with key technocrats at the Uganda Ministry of Finance. Furthermore, the loss of these champions was identified as a major barrier to sustaining momentum in holding the Uganda government to its commitment in the FCTC of progressively increasing taxes on tobacco.

We have lost many champions and as such we have lost the momentum in pushing for increases on taxes on tobacco. In the past we had advocates who were vocal on the issue to consistently raising taxes on tobacco. We no longer have voices talking about increasing taxes about tobacco right now in Uganda. (KI_CSO official_09)

Several of the champions mentioned as having been lost to attrition since 2015 when Uganda passed the tobacco control were civil society organizations (CSO) advocates. It emerged that the Ugandan tobacco tax advocacy coalition particularly CSO advocates were heavily dependent on external grants from foreign funders such as Bloomberg Philanthropies and Gates Foundation for the CSOs on which they depended for their salaries. However, these grants are time-limited and often provided on a competitive basis. Several CSOs engaged in the campaign for higher taxes have ceased being active in tobacco-taxation advocacy when these grants elapsed. Some of the identified champions were Ministry of Health officials who have since passed away or sought alternative employment with a few individuals joining the Uganda government as cabinet ministers. It is not uncommon to find that when tobacco control advocates join government they are constrained in maintaining their original direct advocacy role due to political pressures. In the case of one member of parliament who was the mover of the Uganda anti-tobacco law in parliament, who joined the Ugandan cabinet as a minister, BATU threatened to withdraw contracts for tobacco leaf farmers in the constituency he represents in parliament (Doward 2014).

Participants indicated that the attrition of key champions meant loss of the thread of advocacy on sustained increases in taxes on tobacco advocacy. It was reported that there is a paucity of active tobacco-taxation advocates to hold the Uganda government accountable to the FCTC’s recommended minimum tax threshold of 75%. Participants indicated that the attrition of tobacco-taxation advocates was a contributory factor in tax rates remaining static at 35% particularly for the period between 2017 and 2024.

The academics we interviewed argued that with no sustained advocacy from public health advocates to counter the tobacco industry narrative that raising taxes would cause ‘economic harm’, key decision makers especially in the Ministry of Finance would be more inclined to maintain the status quo in tobacco tax rates.

### Interference in tobacco tax policy by vested interests

Our interviews revealed multiple tactics by industry actors in pushing back against higher taxes on tobacco in Uganda. The identified strategies include what was described as a sustained and well-resourced lobbying campaign targeting policy elite in Uganda. The industry was said to target government officials such as those with decision-making power of setting annual tax rates on tobacco products. Ministry of Finance officials acknowledged that officials from the tobacco industry frequently seek audience with them to make the case for a tax regime ‘that doesn’t kill off the industry’. This is despite calls by the FCTC barring such meetings. In addition, our study unearthed lobbying across both the executive and legislatives arms of government as reported by a participant below:

There is tobacco industry interference at the level of Ministry of Finance where decisions on annual tax rates are decided, interference at the level of parliamentary committees that legislate on annual tax proposals and even within the Ministry of Trade for pushing for ‘friendly’ taxes. (KI_CSO official_11)

It emerged that the Uganda tobacco industry fully exploits the set consultative pre-budget meetings organized by Ministry of Finance and the Ugandan national parliament as part of the processes of legislative consideration of annual national budget estimates which is read in July of every calendar year. It’s at these meetings held between April and May each year that government revenue estimates for the next financial year are set including tax policy proposals such as excise duty on cigarettes. The pre-budget meetings are aimed at garnering feedback on government tax proposals by multiple stakeholders including the local tobacco industry. The meetings are conducted by the Finance Committee of the Parliament of Uganda.

When you are designing a tax policy, you have to consult as much as possible because you could be solving one problem and creating another. Once we propose to adjust tax rates then we have to go to parliament. We have to present adjusted tax rates before committees of parliament such as the ‘Committee on Budget’. Then the responsible committees invite stake holders including Ministry of Health, Civil Society, the industry and have a discussion with them. So it is after those consultative processes that we go to the main house (parliament) and then decisions are made whether to increase or not to increase taxes. (KI_Tax official_04)

Our interviews revealed that BATU has a history of astute selection of former government ministers as board chairs. Those selected were often those thought to have with extensive connections to the highest levels of government in Uganda including those who have are can influence the adoption of favourable tax regimes and ‘could open doors’ with local tax revenue authorities.

To illustrate industry success in pushing back against higher taxes, representatives of CSOs revealed that Uganda had not raised taxes on tobacco between 2017 and 2024 contrary to the FCTC which calls for progressive increases in excise duty to keep up with changes in the economy such as inflation.

The tobacco industry is restless in lobbying against tax increases. If it means seeking audience with the President of Uganda they will go. If it means writing position papers to parliament, they will do that. That's how they fight back. Through lobbying members of parliament, the minister of finance or whoever has authority to approve. (KI_CSO official_09)

Interviews with high-level informants from the Ministry of Finance revealed that radical increases in taxes on tobacco were unlikely in Uganda.

From a taxation perspective, it's difficult to have, for example, a 50% raise abruptly. We can increase gradually. We can only increase by 1%, 2%, something like that. We don't want a high spike in the tax rate because that will impact them (tobacco industry). (KI_Tax official_04)

There was a strong sense particularly among our non-state informants that tobacco tax policy in Uganda was a hotly contested space and was heavily influenced by powerful vested interests and that setting tax rates such as excise duty on tobacco products was driven by political rather than technical considerations and has much to do with ‘elite bargains’.

Setting the rates of taxes on tobacco in Uganda is influenced by powerful groups those with interests to protect and they have friends in very high places and this is at every turn. We are talking about tax policy making, tax administration and policy implementation. (KI_CSO official_01)

In addition, representatives of CSOs engaged in tobacco control advocacy reported that the tobacco industry in Uganda has over the past three decades run a sophisticated media campaign where its ‘talking points’ on the dangers of a ‘repressive’ tobacco tax regime are relayed to the public and political elite in the local media. Participants pointed to examples of a handful of ‘sponsored’ business reporters at the leading government-owned daily in Uganda who consistently push the industry line particularly in the print media on pet subjects such as the risk of illicit trade due to high taxes on tobacco.

Desk reviews revealed that BATU used litigation as pushback against raising taxes on tobacco products. The Uganda government has been sued in courts of law as an industry tactic to maintain the status quo including on excise duty on cigarettes. The industry has vehemently argued that tobacco control taxes infringe on their economic rights through invoking trade and investment laws or global trade treaties to which Uganda is a signatory (Tobacco Tactics 2025).

### Paucity of localized evidence on the effects of higher taxes on tobacco

There was unanimity across multiple participants that the paucity of local evidence simulating the likely impacts of tobacco tax increases in Uganda was a void consistently exploited by the local tobacco industry lobby. It was reported that the Uganda tobacco industry took advantage of the paucity of local data modelling the impact of tobacco taxes to promote its own narrative of the need to maintain the status quo in tobacco tax rates taxes in order not to ‘kill off’ the domestic tobacco industry. Representatives of CSOs reported that the absence of local evidence on the effects of a higher tax regime on tobacco they were bereft of evidence-informed messages for countering the industry narrative that higher taxes would ‘kill off the industry’, would spur illicit trade and hurt Uganda government tax revenue.

Key decision makers in Uganda including those from ministry of finance do not have the correct information on tobacco taxes. They are easily fed with misinformation by the industry. Even members of parliament do not have accurate information on the impact of raising taxes on tobacco and they become easy victims to fearmongering. The minister for finance needs to understand that raising taxes will not lead to closure of the tobacco industry in Uganda but he is not fed with the correct data on tobacco taxation. (KI_CSO representative_02)

Our interviews revealed that the tobacco industry in Uganda used information as a form of power in influencing policy elite in Uganda. Whereas the industry often has ready ‘data’ supporting its policy positions on tobacco tax changes and their effects, there was often no contrary evidence available to legislators and tax authorities in Uganda.

Several participants pointed out that when it comes to taxes on tobacco, public health advocates in Uganda tended to rely on evidence from high-income countries. They mentioned that the available evidence in Africa was from contexts such as South Africa where studies simulating the impact of higher taxes on tax revenues have been done. Local public health researchers we interviewed reported that high-level technocrats such as the Ugandan Ministry of Health who make key decisions on setting rates on excise duty on cigarettes lack expertise in the economics of tobacco taxes and were inclined to believe the industry narrative. They indicated that Uganda has a shortage of specialist economists who have expertise in tobacco taxes such as those who can competently model the effects of raising excise duty on cigarettes on industry revenues, production and consumption.

Representatives of CSOs indicated that the industry had succeeded in ingraining in the minds of Ugandan policy elite and the general public that the current tax rates on tobacco were optimal. They added that in order to dispel this notion, there was a need to generate alternative local evidence countering the industry narrative.

The reality is that there is not much Ugandan data around tobacco taxation. Even if you conduct a systematic review you won’t find sufficient information to do with Uganda. We must admit and confess that we do not have evidence supporting our positions on increasing taxes. Hard data that shows it will not hurt business and government tax income. That can be a problem. It creates inertia. (KI_academic_01)

### Differential tax structure for tobacco products

There was concurrence among multiple participants particularly so among academia that the absence of a uniform tax structure for tobacco products in Uganda complicates efforts aimed at tracking progress on meeting the minimum tax threshold recommended by WHO’s FCTC.

It emerged that Uganda implements a differentiated taxation structure for tobacco products of different types which undermines efforts to hold Uganda government accountable on the tobacco tax and price measures it committed to when it ratified the WHO’s FCTC.

Firstly, participants reported that Uganda charges different excise duty on locally manufactured cigarettes from those which are imported. For instance, although Uganda had not increased taxes on tobacco between 2017 and 2024, in mid-April 2025, the Ugandan Ministry of Finance proposed doubling excise duty on imported cigarettes while it recommended a relatively modest increment of 18% on locally manufactured cigarettes for the financial year 2025/2026 (Daily Monitor 2025). Secondly, whereas excise duty is applied to cigarettes, for other tobacco products such as cigars a different tax regime is applied. The FCTC recommends a ‘single spine’ arrangement or a uniform tax structure for all tobacco products without distinction.

There is a problem with the tax structure for tobacco itself. It is not uniform. It is complex. It has loopholes and gaps. These loopholes have been exploited by the tobacco industry such as having different taxes for different tobacco products which makes it hard to track progress on progressive taxation and to hold government to account for the targets set out in the FCTC. For example, taxes on locally-made cigarettes are too low to effectively curb the use of tobacco products. (KI_CSO representative_04)

CSO advocates observed that taxing locally manufactured cigarettes at a significantly lower rate than imported ones renders locally manufactured cigarettes cheaper and therefore more affordable to particularly younger people and those from the lowest income groups. Participants observed that levying much higher taxes on imported cigarettes was superfluous as not many Ugandans can afford western cigarette brands such as ‘Malboro’ or ‘Benson & Hedges’.

Further still, CSO advocates observed that cigarettes which are taxed as ‘locally manufactured’ incorporates those from almost all neighbouring countries such as Kenya owing to a common market agreement relating to membership in a regional bloc known East African Community altogether this constitutes more than 80% of the cigarettes consumed in Uganda [38].

Taxing locally-made cigarettes lower than imported cigarettes defeats both public health and tax income goals of government. We know that most cigarettes consumed in Uganda are those which are taxed as ‘locally manufactured’ including those from neighboring Kenya which dominates the local market in Uganda. In reality, few Ugandans consume western brands and hence the excise duty should actually target ‘locally-manufactured’ cigarettes if we to have real impact. (CSO advocate-04)

Participants further pointed out that the FCTC recommends that the tax be levied on the retail price of tobacco. However, in Uganda the tax is levied per 20 sticks of cigarettes hence the tax share of the final price of cigarettes is low thereby undermining the role of taxation as a price measure for reducing tobacco consumption.

## Discussion

We set out to understand barriers to raising taxes on tobacco in Uganda in line with WHO’s recommendation that tax constitute 75% of the retail price of tobacco products. We found that the tobacco industry narrative suggesting that raising taxes beyond the current 35% of excise duty on cigarettes would bring economic harm was deeply entrenched among policy elite in Uganda. Interviews with policy elite revealed that they bought into the industry’s narrative of the danger of raising taxes that ‘kill off the industry’ and therefore loss of government tax revenue, loss of jobs along the tobacco value chain in Uganda and that it would exacerbate illicit trade through cross-border smuggling. Our study unearthed the tendency of the Uganda tobacco industry to create an information asymmetry where policy elite did not have alternative data to that provided by the tobacco industry. Our study reveals that the industry has interfered in tobacco tax policy in Uganda using multiple tactics including through exploiting annual stakeholder meetings with legislators and ministry of finance officials for garnering feedback on tax proposals in national budget estimates. We found that Uganda operates multiple tobacco tax regimes contrary to WHO recommendations of adopting a ‘single spine’ tax structure which impedes attainment of public health agendas of reducing consumption of tobacco. There was a strong sense among participants that tobacco tax policy making in Uganda was a hotly contested space and that the process was heavily influenced by powerful vested interests. There was a widely held perception that that setting tobacco tax rates in Uganda was shaped more by political economy considerations than technical criteria and that it was largely an outcome of a ‘tacit agreement’ between the Uganda tobacco industry and policy elite.

### A political settlements analysis perspective

Utilizing PSA lenses we find that tax rates on tobacco in Uganda are influenced through protracted industry lobbying of policy elite in Uganda. Taxes on tobacco in Uganda appeared to have been reached through an implicit political settlement reached between the industry and Ugandan policy elite (Kelsall 2018, Khan 2018). Participants alluded to an invisible ‘equilibrium’ beyond which policy elite in Uganda were convinced by actors in Uganda’s dominant tobacco company, through an intense lobbying campaign, that raising taxes would result in ‘killing off the industry’ which would culminate in loss of tax income to the national government (Ulucanlar et al. 2016). We observe the use of power in terms of ownership of ‘evidence’ on the impact of raising taxes whereby the tobacco industry in Uganda owned and controlled ‘evidence’ on the impact of raising taxes on tobacco and the use of threats of litigation against the national government for violating ‘economic and trade rights’ of the industry if it raised taxes on tobacco in a way that was detrimental to the industry. This has been termed ‘the policy dystopia model’ (Ulucanlar et al. 2016). Previous studies have alluded to the occasional clashes between trade rights and public health objectives (Blouin 2007). We note the industry in Uganda exhibited superior organizational capability of engaging in sustained lobbying of key decision makers for favourable tax regimes as well as economic resources such as in attending pre-budget meetings on annual basis. In this regard, Khan (2018) has alluded to ‘the capability of an individual or group to engage and survive in conflicts’ as being ‘a product of diverse factors, including economic resources, organizational capacity and the ability to absorb costs’. With respect to the ‘the balance of power’ between contending interest groups we observe that the contest between tobacco control advocates and the tobacco industry regarding the framing of tobacco taxes we find a power imbalance (Kelsall 2018, Khan 2018). While the tobacco industry in Uganda dominated information flow to policy elite (Ulucanlar et al. 2016), tobacco control advocates lacked resources for generating alternative credible evidence. We note the attrition of ‘champions’ among Ugandan advocates campaigning for higher taxes on tobacco while we observe a sustained campaign by the tobacco industry for maintaining the status quo in tobacco tax levels particularly between 2017 and 2024. Elliot et al. (2020) have identified ‘policy championing’ as a key influencing factor in the uptake of health taxes in a scoping review they conducted.

In addition, with respect to the PSA notion of ‘mobilization capability’ we observe that the tobacco industry demonstrated superior ability specifically with regard to its ability to magnify the constituencies it purports to represent such as tobacco leaf farmers and retail and wholesale dealers in tobacco products, the continued promotion of its ‘large tax payer’ status as a part of its ‘elite bargaining’ for a more favourable ‘settlement’ with respect to setting tax rates. Furthermore, the industry used litigation as a ‘hard tactic’ in its arsenal of strategies and also leveraged its networks with officials from the Uganda Ministry of Trade in pushing for ‘friendly’ taxes that supposedly promote trade and local industry.

### Implications for monitoring tobacco industry interference

Although the FCTC bars physical meetings between the tobacco industry and government officials, in this study it was revealed that policy elite such as Ministry of Finance officials, government tax authorities and legislators frequently interface with the tobacco industry lobby under multiple guises including multi-stakeholder preparatory meetings for the national budget. In addition, interviews with government officials responsible for setting tobacco tax policy revealed constant engagement with industry actors which was shown through government officials rehearsing ‘talking points’ from the industry such as on illicit trade and ‘economic harm’ of increasing taxes on tobacco (Smith et al. 2013). Our study adds crucial in-depth insights into tobacco industry tactics of pushing back against raising taxes in a low-income country context thereby improving our understanding of potential pathways for addressing industry interference in tobacco tax policy (Ulucanlar et al. 2016). Monitoring industry interference in tobacco control is a core tenet of the FCTC. Our findings point to the need for policy and programming interventions for devising effective strategies for monitoring industry interference in tobacco tax policy particularly in low-income countries which are dependent on tax income from tobacco (Ulucanlar et al. 2016, Delobelle 2019, Reeve and Gostin 2019). Reeve and Gostin (2019) have called for a ‘comprehensive approach to reducing inappropriate industry influence requires that rigorous conflicts of interest rules must be woven into—and supported by—broader governance structures’.

Our study suggests an information asymmetry between Ugandan policy elite and the tobacco industry regarding the effects of raising tobacco tax whereby the later wielded power over presumed evidence on the subject. Information was used as a form of power by the industry lobby which took advantage of the paucity of alternative local empirical data to that provided by the tobacco industry in terms of simulating the effects of increasing taxes on tobacco in Uganda. Tobacco control advocates highlighted the lack of non-industry-funded local evidence as a major hindrance in their attempts to counter industry narratives. Our study points to the need to build a local evidence base on the effects of raising taxes on tobacco under multiple scenarios to inform policy decisions without reliance on industry-leaning data. Hoe et al. (2021) found that the availability of local evidence in Philippines and Ukraine enabled those countries adopt higher taxes on tobacco.

Our study extends the application of the PSA framework to tobacco tax policy analysis thereby expanding the repertoire of PEA approaches in this space. Future research should explore further the notion of ‘tacit agreements’ between policy elite and the tobacco industry lobby. This is particularly relevant in low-income countries where national governments are more dependent on tax income from institutional ‘large’ tax payers that include the tobacco industry. Our study unearthed Uganda tobacco industry tactics of exploiting annual national budget stakeholder meetings, organized by the Uganda Ministry of Finance, as a platform for lobbying policy elite to secure favourable tobacco tax policies. In this study, it appeared that through its ‘corporate power and corporate involvement’ (Holden and Lee 2009), the tobacco industry in Uganda appeared to provide overt indication to policy elite in Uganda as to which tax thresholds were acceptable. This re-enforces the PSA notion of ‘bargaining outcomes’ (Kelsall 2018, Khan 2018) between national governments interested in protecting tax revenues and tobacco interests pushing for ‘optimal’ tax levels which is an under-explored notion in understanding barriers to uptake of health taxes in LMICs and is deserving of further empirical scrutiny in future research.

Our study adds to the literature on the political economy of health taxes in LMICs. Overall, there is concurrence around the notion of the tobacco industry’s success in framing health taxes in their favour. A study in four countries in Sub-Saharan Africa found barriers in the use of health taxes as a fiscal measure in the response to NCDs due to ‘challenges from entrenched economic policy paradigms for industry-led economic growth’ (Thow et al. 2021), a similar finding in Nigeria was reported on ‘policy-framing efforts to undermine tobacco control policies’ by the tobacco industry (Akin-Onitolo and Hawkins 2022). A scoping review of health taxes in LMICs found that ‘negative framing and retaliation by industry’ impeded the effectiveness of health taxes with respect to alcohol, food and drink (Elliot et al. 2020). In this study we found that the tobacco industry in Uganda used the livelihoods of tobacco leaf farmers to overstate its importance to the Uganda economy an industry tactic identified in studies in Zimbabwe and Mozambique (Lencucha et al. 2020).

An important finding of our study is that the Uganda Government utilized tobacco tax policy as an additional fiscal measure for protecting domestic industries. Our findings revealed that while the Uganda government proposed to double excise duty on imported cigarettes, it only proposed a modest increment of 18% for locally manufactured cigarettes. Although tobacco tax policy has been linked with the dual goals of increasing government tax revenues and promoting public health, the use of taxes on tobacco as a tool of protectionism has not received much empirical attention which warrants further research and appropriate tobacco control strategies.

### Limitations

Our study had some limitations. While we endeavoured to interview a diverse set of relevant stakeholders influential in tobacco tax policy making in Uganda, we did not interview representatives of the tobacco industry in Uganda which would have made for a more rounded analysis. Furthermore, we used a retrospective approach of exploring drivers of tobacco tax policy by ‘looking back’ over the past 5–7 years thereby relying on participants’ memories. The potential limitation in this approach is recall bias. However, our study had multiple strengths. These include the use of triangulation in our data sources such as the use of key informant interviews and desk reviews of published reports on tobacco industry tactics in Uganda. Furthermore, we applied a political economy lens which helped unravel the complex economic and political considerations or the ‘elite bargains’ that underpin tobacco tax policy in Uganda.

## Conclusion

We found that the diminished presence of Ugandan CSO in advocating for higher tobacco taxes in Uganda left a vacuum that saw the ascendance of a pro- tobacco industry narrative around maintaining the status quo in the tobacco tax regime in Uganda. Our findings highlight the need for strengthening civil society advocacy in order to sustain the momentum on raising tobacco taxes in Uganda.

## Data Availability

The datasets generated during and/or analyzed during the current study are not publicly available due to ethical reasons but are available from the corresponding author on reasonable request.

## Reflexivity statement

The group of authors includes researchers at the early career (H.Z., E.S., and R.S.), mid-career (L.P.) and senior (F.S.) career stages, from Africa (Uganda) and North America (L.P.). This represents a good balance in terms of seniority. We have one female author on the team and three are male. We received funding for this study from WHO Alliance for Health Policy and Systems Research and we have included this fact in the declarations section. Although this study was externally funded, it relays local stakeholder perspectives on the political economy of tobacco taxation in Uganda a low-income country.
